# Volumetric Modulated Arc Therapy Craniospinal Irradiation Utilizing a Vertebral Body Sparing Approach: An Alternative Approach to Improve Access and Minimize Toxicity

**DOI:** 10.1016/j.adro.2023.101424

**Published:** 2023-12-14

**Authors:** Haley K. Perlow, Jennifer K. Matsui, Ashlee Ewing, Catherine Cadieux, Khaled Dibs, Rituraj Upadhyay, Dukagjin M. Blakaj, Sasha J. Beyer, Evan M. Thomas, John C. Grecula, Raju R. Raval, Joshua D. Palmer

**Affiliations:** a Department of Radiation Oncology, The Ohio State University Wexner Medical Center, Columbus, Ohio; b Ohio State University School of Medicine, Columbus, Ohio

## Abstract

**Introduction:**

Craniospinal irradiation (CSI) is indicated for adult patients diagnosed with leptomeningeal disease (LMD). Proton-based vertebral body sparing (VBS) CSI has been explored with pediatric patients to minimize hematologic toxicity; however, utilization of VBS in an adult population is limited. A recent phase II trial has shown efficacy of proton-based CSI to treat non-small cell lung and breast cancer with LMD. We hypothesize that VBS CSI using volumetric modulated arc therapy (VMAT) could also effectively reduce dose to vertebral bodies and surrounding organs at risk, minimizing toxicity for adult patients with LMD and comparing favorably to proton-based CSI.

**Methods and Materials:**

Consecutive patients with LMD received VMAT VBS CSI, 30 Gy in 10 fractions, as a part of a prospective registry. Full VMAT arcs for the brain fields matched to 2 spine isocenters for the upper and lower spine were created using limited posterior arcs. To further decrease the vertebral body dose, an avoid entry and exit contour was created. Acute toxicity data were collected using Common Terminology Criteria for Adverse Events v5.

**Results:**

Ten adult patients were treated in this cohort. One patient experienced grade 2 neutropenia with the remaining 9 experiencing grade 1 hematologic toxicity. Three patients experienced grade 2 gastrointestinal toxicity with the remaining 7 experiencing grade 1 nausea. No patient experienced grade 3+ toxicities in this cohort. One patient experienced a 5-day delay in systemic therapy initiation due to neutropenia; otherwise, all patients planned for systemic therapy started without delay.

**Conclusions:**

In this study, VMAT VBS CSI led to acceptable toxicity compared with patients treated with proton CSI on a phase 2 clinical trial. Given its promising early results, future prospective evaluation of the technique is warranted.

## Introduction

Craniospinal irradiation (CSI) plays a critical role in the treatment of tumors that have a high incidence of leptomeningeal dissemination. CSI delivered with photons can result in significant acute toxicity, with historical rates of grade 3+ hematologic toxicity around 30%.^1^^,^^2^ As a result, proton-based CSI has emerged as a standard option for pediatric and adult patients for many brain tumors including medulloblastoma, germ cell tumors, pineoblastoma, and ependymoma.^3^^,^^4^ A recently published phase II trial of proton CSI (pCSI) versus involved field radiation has shown how pCSI can improve central nervous system progression-free survival and overall survival for patients with non-small cell lung cancer and breast cancer with leptomeningeal disease (LMD), albeit with similar hematologic toxicity compared to involved field radiation.^5^ However, proton therapy is not a widely available treatment modality.^6^ We propose that vertebral body sparing (VBS) CSI using volumetric modulated arc therapy (VMAT) will effectively reduce vertebral body dose while minimizing dose to surrounding organs at risk (OAR) for adult patients with LMD, which in turn may produce a similar toxicity profile compared with pCSI.

## Methods and Materials

**Figure 1:**
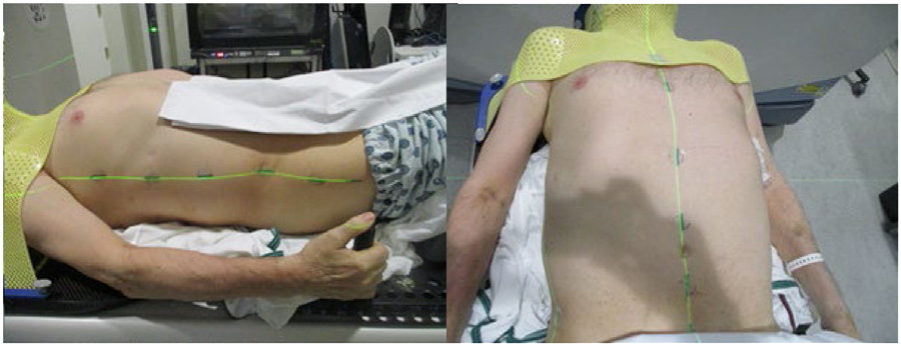
Example of towel involvement during computed tomography simulation scan.

Consecutive patients with LMD received VMAT VBS CSI, 30 Gy in 10 fractions, as a part of a prospective registry that treated patients from 2017 to 2022. In some instances, patients were simulated with a lengthwise-folded towel placed under their arms from above the elbow to the wrist to comfortably elevate the arms from the table (Fig. 1). The folded towels result in enough elevation to position the arms further out of the field to avoid excess dose from the limited posterior arcs. Full VMAT arcs for the brain fields matched to 2 spine isocenters for the upper and lower spine were created using limited posterior arcs. All beams were coplanar, and beam angles used are 181 to 255 and 110 to 179. Autofeathering in the optimizer and robust optimization was used for field overlap, and all 3 plan isocenters (brain, upper spine, lower spine) were optimized in the same plan with the full planned target volume (PTV) CSI, then split into separate isocenters after optimization for imaging purposes at delivery. Eclipse, Acuros 16.1 was used for treatment planning. The PTV was created with margins of 3 mm uniformly around the brain contour and 7 to 10 mm around the spinal canal. To further decrease the vertebral body dose, an avoid entry and exit contour was created. Avoidance structures in the optimization are used to block the radiation beam when avoidance structures are both in front of and behind the target and can block radiation pixel-by-pixel in a fluence plane. This avoidance structure can be activated by setting the structure type to entry or entry + exit for the selected structure; if the radiation through a certain fluence pixel hits the avoidance structure before or after hitting the target, radiation is blocked through that fluence pixel. This structure was a margin on the PTV anteriorly designed to carve dose out of the vertebral bodies while still maintaining coverage to the PTV. For image guidance, orthogonal imaging for straightening and subsequent cone beam computed tomography scans were taken daily. All patients were offered ondansetron as needed to control treatment-related nausea. Physician reported acute toxicity data for VMAT VBS CSI was collected using Common Terminology Criteria for Adverse Events v5 and was defined as toxicity occurring within 30 days of treatment conclusion. Leukopenia was defined as a white blood cell count <3.99 K/uL. Extracraniospinal axis disease control was defined as stable disease outside the craniospinal axis or absence of disease.

**Figure 2:**
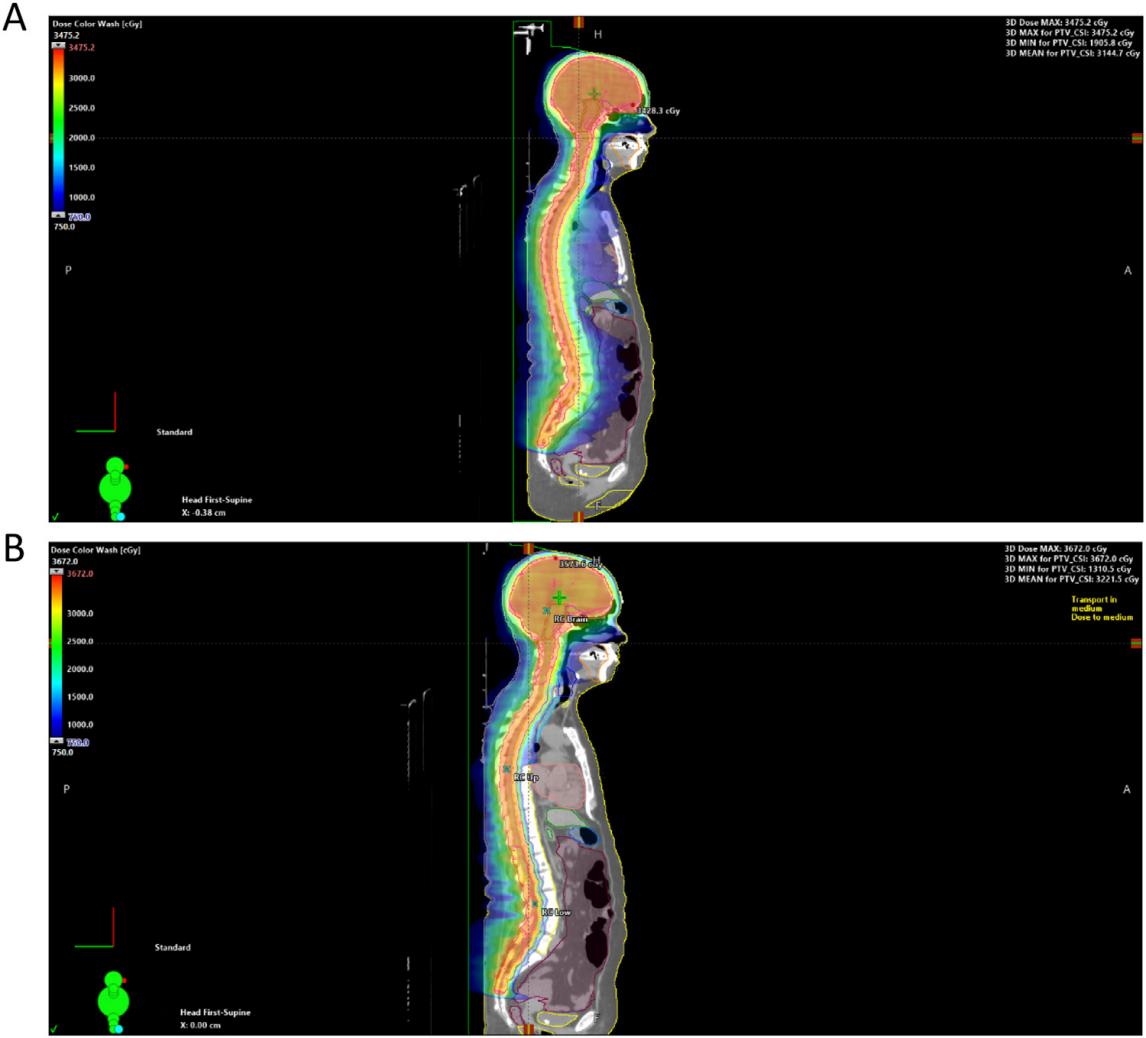
Plan comparison of volumetric arc therapy vertebral body sparing craniospinal irradiation (CSI) and standard CSI plan. (A) Standard CSI plan. (B) Volumetric arc therapy CSI plan.

An additional analysis evaluated 10 patients initially treated with a variety of VMAT and intensity modulated radiation therapy (IMRT) treatment techniques that did not involve sparing the vertebral bodies. For these plans, a 3 isocenter treatment technique is typically needed to cover the entire PTV. Three VMAT arcs were used for the brain isocenter, and the patients with IMRT had single posterior anterior (PA) IMRT spine fields, while the patients’ planned VMAT had the small posterior arcs designed to spread out dose and create fewer hot spots. The original VMAT plans focused on decreasing dose to anterior structures including the esophagus, larynx, and bowel. The IMRT and VMAT plans were optimized with all isocenters together in the treatment planning system using autofeathering to create more robust plans considering the shifts to each isocenter. The ideal overlap between isocenters is between 5 to 10 cm. These patients were then retrospectively replanned using VMAT VBS CSI (example in Fig. 2). The dosimetric differences of these plans were compared for each patient.

## Results

Ten adult patients were treated with VMAT VBS CSI in this cohort (Table 1). Half of the treated patients were female. The median age was 57 years old (range, 20-71). Forty percent of patients had a primary breast malignancy. Six patients had a Karnofsky Performance Status of at least 70. Only 2 patients had extracraniospinal metastases when evaluated for CSI, but all 10 had extracraniospinal axis disease control. Most patients (70%) received prior brain radiation, with 3 patients receiving prior stereotactic radiosurgery, 1 patient receiving prior whole brain radiation therapy, and 3 patients with glioblastoma who received partial brain radiation. For the patient with prior whole brain radiation, repeat treatment was necessary due to rapid progression of significant LMD.

**Table 1:** Patient characteristics

Characteristic	N = 10
Sex	
Male	5 (50%)
Female	5 (50%)
Age (median, IQR)	57 (35, 62)
Primary tumor	
Breast	4 (40%)
Brain	3 (30%)
Lung	2 (20%)
Cervix	1 (10%)
Karnofsky performance status	
100-90	2 (20%)
80-70	4 (40%)
60-50	2 (20%)
40	2 (20%)
Extracraniospinal disease control	
Controlled	10 (100%)
Uncontrolled	0 (0%)
Extracraniospinal metastases	
Present	2 (20%)
Absent	8 (80%)
Previous brain radiation	
Yes	7 (70%)
No	3 (30%)
Completed CSI	
Yes	9 (90%)
No	1 (10%)

*Abbreviations*: CSI = craniospinal irradiation; IQR = interquartile range.

For all ten patients, complete blood counts were routinely drawn during and after treatment, but differentials were not always available. One patient experienced grade 2 neutropenia, with the remaining 9 experiencing grade 1 hematologic toxicity including 3 patients with grade 1 pancytopenia and 6 patients with grade 1 thrombocytopenia (Table 2). Three patients experienced grade 2 gastrointestinal toxicity (grade 2 nausea, grade 2 esophagitis, grade 2 esophagitis/grade 2 diverticulitis) with the remaining 7 experiencing grade 1 nausea. No patient experienced grade 3+ toxicities in this cohort. Only 1 patient did not complete all 10 radiation treatments. This patient had a poor performance status (Karnofsky Performance Status of 40) and completed only 8 treatments before deciding to transition to hospice after experiencing persistent grade 1 fatigue and nausea. Overall, 4/10 patients proceeded with systemic therapy immediately after CSI, with 1 additional patient proceeding after a short delay (patient initially declined systemic therapy). One patient experienced a 5-day delay in systemic therapy initiation due to neutropenia; otherwise, all patients planned for systemic therapy started without delay. The systemic therapies initiated were either bevacizumab, trastuzumab, or carboplatin/etoposide. For all 10 patients in the study, CSI was initiated upfront after diagnosis of LMD and was not delayed for systemic therapy.

**Table 2:** Treatment toxicity

	Acute toxicity				
Toxicity	1	2	3	4	Total
Fatigue	4	1	0	0	5 (50%)
Vertebral fracture	1	0	0	0	1 (10%)
Cognitive decline	1	0	0	0	1 (10%)
Ataxia	1	0	0	0	1 (10%)
Weight loss	2	3	0	0	5 (50%)
Alopecia	2	0	0	0	2 (20%)
Vision change	0	2	0	0	2 (20%)
Leukoencephalopathy	0	1	0	0	1 (10%)
Nausea/Vomiting	7	1	0	0	8 (80%)
Diverticulitis	0	1	0	0	1 (10%)
Esophagitis	0	3	0	0	3 (30%)
Thrombocytopenia	9	0	0	0	9 (90%)
Leukopenia	3	1	0	0	4 (40%)
Pancytopenia	3	0	0	0	3 (30%)

Patients planned for VMAT VBS CSI displayed dosimetric improvements compared with patients receiving CSI without VBS (Fig. 2). The vertebral body mean dose was reduced by 33% with VBS CSI (Table 3). The small bowel and esophagus mean doses were decreased by 46% and 67%, respectively, with VMAT VBS CSI. The small bowel and esophagus maximum doses were decreased by 13% and 35%, respectively.

**Table 3:** Comparing volumetric arc therapy (VMAT) vertebral body sparing (VBS) craniospinal irradiation (CSI) with standard plans for 10 patients

Dosimetry	Number or Percent
Vertebral body mean (% of prescription dose)	
Standard plan	91.7%
VMAT VBS CSI	61.5%
Average reduction	33%
Small bowel mean (cGy)	
Standard plan	720.3
VMAT VBS CSI	386.0
Average reduction	46%
Small bowel maximum (cGy)	
Standard plan	2415.4
VMAT VBS CSI	2106.6
Average reduction	13%
Esophagus mean (cGy)	
Standard plan	1741.1
VMAT VBS CSI	570.4
Average reduction	67%
Esophagus maximum (cGy)	
Standard plan	2699.8
VMAT VBS CSI	1757.0
Average reduction	35%

## Discussion

These data show how VMAT VBS CSI can be used to minimize dose to surrounding OARs and vertebral bodies and minimize treatment related toxicities for adult patients with LMD. Yang et al's phase II trial of pCSI for adult patients with LMD showed that there was still hematologic toxicity from treatment related to vertebral body dose.^5^ In this phase II study, 10%, 19%, 65%, 7%, and 7% of patients experienced grade 2+ anemia, leukopenia, lymphopenia, neutropenia, and thrombocytopenia, respectively. Additionally, 5% and 4% of patients experienced grade 2+ nausea or vomiting, respectively. Esophagitis or dysphagia were not documented in the phase II trial. In our cohort of patients receiving VMAT VBS CSI, where was only 1 episode of grade 2 neutropenia; all other hematologic toxicities were grade 1. Three patients experienced grade 2 gastrointestinal toxicities. There was no grade 3+ toxicity in our cohort. One limitation is that absolute lymphocyte count was not collected for all patients, which may be notable because even low doses of radiation may affect lymphocyte count. Overall, the toxicity profile of VMAT VBS CSI compares favorably to pCSI.

## Conclusion

VMAT VBS CSI is an effective technique to reduce dose to surrounding OARs and vertebral bodies. In this study, VMAT VBS CSI led to acceptable toxicity compared with patients treated with pCSI on a phase 2 clinical trial. In addition, we showed the degree of reduction in dose to the vertebral body, small bowel, and esophagus when comparing VMAT VBS CSI to conventional VMAT and IMRT CSI plans. An NRG phase 3 clinical trial may be developed to evaluate the efficacy of pCSI for patients with LMD. However, these data show how VMAT VBS CSI may be an acceptable alternative for centers without proton therapy capabilities. Given its promising early results, future prospective evaluation of the technique is warranted.
